# Effects of Skeletal Muscle Hypertrophy on Fat Mass and Glucose Homeostasis in Humans and Animals: A Narrative Review with Systematic Literature Search

**DOI:** 10.1007/s40279-025-02263-w

**Published:** 2025-06-27

**Authors:** Tim Havers, Steffen Held, Martin Schönfelder, Stephan Geisler, Henning Wackerhage

**Affiliations:** 1 Exercise Biology Group, School of Medicine and Health, Technical University of Munich, Munich, Germany; 2 Department of Fitness and Health, IST-University of Applied Sciences, Düsseldorf, Germany; 3 Department of Sport and Management, IST University of Applied Sciences, Düsseldorf, Germany

## Abstract

Overweight, obesity, and type 2 diabetes mellitus are metabolic health problems and diseases that affect billions of people worldwide. Studies in animals and humans suggest that stimulating global muscle hypertrophy could be a treatment for these diseases, as some key studies suggest that stimulation of global muscle hypertrophy commonly reduces fat mass and improves glucose homeostasis. To analyse the effect of muscle hypertrophy on fat and glucose homeostasis in more detail, we systematically searched the literature and quantitatively analyzed 122 studies (humans: *n* = 99; animals: *n* = 23). This analysis reveals that a 1.9–3.3% increase in global muscle mass in humans is associated with 4.1 ± 5.8% lower fat mass, a mean relative reduction in HbA1c of 4.1 ± 4.6% from baseline, and a reduction of fasting glucose concentrations by 5.8 ± 7.3% in studies lasting 2 weeks to 3 years. In the animal studies analyzed, the researchers increased muscle mass by transgenesis, drugs, or resistance training by 17.7 ± 18.4%. This increase of muscle mass was associated with 23.7 ± 22.3% less fat mass. In the second part of this review, we discuss mechanisms by which muscle hypertrophy can affect fat mass and glucose homeostasis. We also discuss the potential use of hypertrophy-focused resistance training and muscle hypertrophy-stimulating drugs as treatments for people with overweight, obesity, and type 2 diabetes.

**Supplementary Information:**

The online version contains supplementary material available at 10.1007/s40279-025-02263-w.

## Key Points

**Table:** 

The stimulation of global muscle hypertrophy in humans and animals typically reduces fat mass and improves glucose homeostasis.
Clinical trials should investigate whether hypertrophy-oriented resistance training, drugs, and other interventions cause fat loss and improve glucose homeostasis in people who are overweight, obese, or have type 2 diabetes mellitus.
Body weight or BMI are poor variables for fat loss interventions that also affect muscle mass. Therefore, weight loss trials should measure not only body mass but also muscle and fat mass, e.g., by bioimpedance or DEXA measurements.

## Introduction

Obesity and type 2 diabetes mellitus are the most common metabolic diseases worldwide. In 2016, 1.9 billion adults were overweight, of whom 650 million were obese, out of a global population of almost 8 billion [1]. Treating overweight and obesity is important as these are risk factors for many other diseases, such as type 2 diabetes [2]. Obesity is currently treated with lifestyle interventions, including calorie-restricted diets, physical activity, and behavioral therapy. In addition, anti-obesity drugs [3], such as the GLP-1 receptor agonist semaglutide (trade names: Wegovy and Ozempic) are effective in causing major weight loss in patients with obesity through the suppression of appetite and food intake [4, 5]. However, while these drugs mainly cause patients to lose fat mass, skeletal muscle is lost as well, which is especially a concern in elderly patients [6]. Patients who are severely obese or who have not responded sufficiently to other therapies can undergo bariatric surgery as a last resort [7].

In 2021, 529 million people were living with diabetes mellitus worldwide. Over 95% of these patients had type 2 diabetes mellitus [8]. Like obesity, lifestyle modification is the first-line treatment. Lifestyle modification includes a healthy and hypocaloric diet to limit hyperglycaemic periods and regular exercise [9]. To improve glycemic control, patients are additionally prescribed drugs that stimulate insulin secretion or improve insulin sensitivity, and in more severe cases, insulin therapy [10]. In recent years, additional drug classes such as sodium-glucose co-transporter 2 (SGLT2) inhibitors and glucagon-like peptide-1 receptor agonists (GLP-1 RAs) have been introduced. They act through several mechanisms, such as increasing glucose excretion via urine, delaying gastric emptying, reducing appetite, and weight loss [4, 11]. In some cases, an aggressive therapy that leads to substantial weight loss can cause type 2 diabetes mellitus remission [12].

Evidence suggests that skeletal muscle is a key organ for body composition and glucose homeostasis. Skeletal muscle is the primary site of insulin-stimulated glucose uptake after a meal. Approximately 75% of glucose disposal following a meal occurs in skeletal muscle, whereas adipose tissue contributes only around 5%, despite also expressing the insulin-responsive glucose transporter GLUT4 [13–15]. Beyond GLUT4, skeletal muscle expresses several other glucose transporters, such as GLUT1, GLUT3, GLUT6, GLUT10, and GLUT12, albeit at lower abundance [16]. While their overall contribution to glucose uptake is small, some of these transporters mediate insulin-independent glucose uptake and are responsive to muscle contraction [16, 17].

**Fig. 1:**
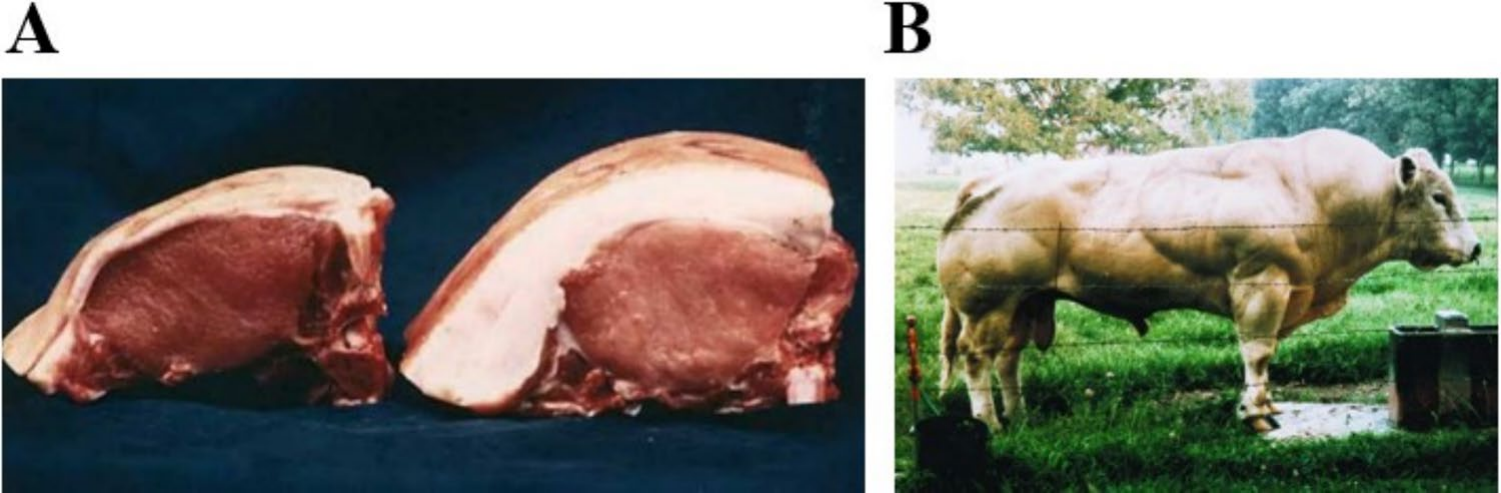
Muscle building and fat lowering effects of repartitioning agents and myostatin inhibition. **A** Cross-sections at the 10th rib of pigs [19]. Left represents a pig treated with 3 mg clenbuterol/kg feed for 4 weeks, plus a 3-week course of daily injections of 0.1 mg recombinant porcine growth hormone/kg liveweight. Right represents a typical control animal. **B** Belgian blue bull showing double muscling and low fat mass. These animals have “natural” mutations of the muscle mass-inhibiting *Mstn* gene. [Copyright (1997) National Academy of Sciences] [20]

There is evidence that greater skeletal muscle mass results in fat loss and improved glucose homeostasis. This is well known in agriculture. The fat-reducing effect of muscle hypertrophy is known as “repartitioning.” For example, livestock farmers in countries such as the USA use β2-adrenergic agonists to “repartition” (i.e., increase muscle mass/meat and reduce fat mass) animals such as cattle or pigs prior to slaughter [18, 19]. The muscle building and fat lowering effects of such treatments are illustrated in Fig. 1.

The effects of global muscle hypertrophy on body fat and glucose homeostasis are especially obvious in transgenic animals, such as mice, where the transgene induces global muscle hypertrophy. For example, myostatin knockout mice [21] or mice in which constitutively active Akt1 is expressed only in skeletal muscle [22] have not only more skeletal muscle mass but also less fat mass and improved glucose homeostasis when compared with wild-type controls. For example, Izumiya et al. found that induction of a constitutively active Akt1 transgene in mice on a high-fat/high-sugar diet resulted in an increase in gastrocnemius muscle mass from approximately 190 mg to approximately 280 mg, and a reduction in inguinal and subcutaneous adipose tissue depots by almost 50%. This treatment also normalized blood glucose levels from approximately 180 mg/dL to approximately 140 mg/dL and decreased serum insulin levels from approximately 1.6 ng/mL to 0.7 ng/mL compared with control mice [22]. Other studies report similar metabolic effects of global muscle hypertrophy in humans. For example, when patients with overweight or obesity and type 2 diabetes were injected every 4 weeks for 48 weeks with either 10 mg/kg of the myostatin receptor inhibitor bimagrumab versus placebo, lean mass (a proxy for muscle mass) increased by 1.70 kg (80% CI: 1.14 to 2.26 kg), fat mass dropped by 7.49 kg (80% CI: −8.33 to −6.64 kg), and HbA1c dropped by 0.76% (80% CI: −1.05 to −0.48%) [23]. Moreover, testosterone administered to men with hypogonadism and obesity not only induces skeletal muscle hypertrophy but also reduces body weight and fat mass [24]. Taken together, the results of these studies suggest that stimulating global muscle hypertrophy causes organisms to lose fat and improves impaired glucose homeostasis. The aim of this review is to systematically summarize published data on the effects of skeletal muscle hypertrophy on fat mass and glucose homeostasis. We will first discuss treatments and genes that can induce skeletal muscle hypertrophy. Second, we will summarize the results of a systematic literature search on the effects of muscle hypertrophy on fat mass and glucose homeostasis in animals and humans. Third, we will discuss potential mechanisms that can explain the associations between muscle mass, fat mass, and glucose homeostasis. Finally, we will discuss clinical applications.

However, a methodological challenge arises when analyzing interventions that aim to increase muscle mass through resistance training. Exercise itself, independent of muscle hypertrophy, can potentially reduce fat mass and improve glucose homeostasis by increasing energy expenditure, enhancing insulin sensitivity, and stimulating glucose uptake via contraction-mediated pathways [25–27]. As such, it can be difficult to disentangle the direct metabolic effects of increased muscle mass from those of the physical activity required to achieve it. This distinction is essential when interpreting data and will be addressed in this review.

## Genes, Drugs, and Methods that Cause Skeletal Muscle Hypertrophy

Skeletal muscle hypertrophy is defined as an increase in muscle mass or cross-sectional area. Muscle mass and cross-sectional area can be measured on the level of the whole muscle or on the level of muscle fibers [28, 29]. In an average woman or man, the over 600 skeletal muscles contribute ≈ 30–40% to body mass, with extremes ranging from 20 to 50% [27]. This demonstrates that muscle mass varies greatly in the human population. This is due to a large variation of the number of muscle fibers and the size of these muscle fibers within a given muscle. For example, Lexell et al. quantified fiber number and measured fiber size in the vastus lateralis muscles of accident victims. They reported that the mean cross-sectional area of type 2 fibers ranged from 2142 ± 300 to 5535 ± 1310 µm^2^ [30]. For comparison, type 2 fibers in male bodybuilders can reach a mean size of 9600 µm^2^ [31]. Similarly, the total number of fibers counted in the same vastus lateralis cross-sections varied widely, ranging from 393,000 to 903,000 muscle fibers in young men [30].

Transgenic mouse studies suggest that muscle size is a polygenic trait, as there are at least 47 genes, whose gain or loss-of-function results in muscle hypertrophy in mice [32]. It is likely that DNA sequence variations of many of these mice also contribute to the variation of muscle mass in humans.

In addition, skeletal muscle hypertrophy can be stimulated by both physiological and pharmaceutical interventions:

1. In humans, the most common intervention to induce muscle hypertrophy is progressive resistance training. Both high-load (> 60% of one-repetition maximum, 1RM) and low-load (< 60% 1RM) protocols can induce hypertrophy of a similar magnitude [33]. Research into resistance training variables shows that load, volume, frequency, and rest intervals, influence the amount of skeletal muscle hypertrophy induced [33–35].
2. In animal models, mTORC1 activation either by animal-specific resistance training, IGF-1 [36], or Akt1 expression [37] causes muscle hypertrophy.
3. In animal models, the loss of myostatin signalling, either through the loss of myostatin itself [38, 39] or by targeting the myostatin receptor with antibodies such as bimagrumab causes muscle hypertrophy [23].
4. β2-adrenergic agonist such as salbutamol [40], clenbuterol [41], and ractopamine [42] promote muscle hypertrophy in animal models. Athletes have misused these drugs, although their use in humans is off-label, controversial, or prohibited in sports owing to their classification as doping agents and concerns over adverse effects and ethical issues [43].
5. Testosterone [44] and related anabolic androgenic steroids [45] increase muscle mass in both animals and humans. While they can be used therapeutically e.g., in patients with hypogonadism, their use in sport is considered doping and is prohibited [46, 47].

In summary, skeletal muscle mass, the number of muscle fibers and their size within a given muscle vary greatly in the human population. Skeletal muscle hypertrophy and other health benefits can be induced by resistance training, which is why resistance training is recommended as a health intervention e.g., by the World Health Organization (WHO) [48]. In addition, drugs that target β-adrenergic receptors, testosterone, and myostatin signaling cause muscles to hypertrophy. Although these drugs are effective, they can cause side effects, as is the case with other medications used to treat obesity and type 2 diabetes.

## Systematic Literature Analysis to Find Out Whether Muscle Hypertrophy Is Associated with Altered Fat Mass and Glucose Homeostasis

One issue is that, so far, we have “cherry picked” studies that report fat and glucose-lowering effects of muscle hypertrophy, implying a risk of bias. To avoid a biased analysis of the metabolic effects of muscle hypertrophy, we decided to conduct a systematic search of the relevant literature on the effects of muscle hypertrophy on fat mass and glucose homeostasis in both humans and animals.

The main findings are summarized below, with additional methodological details provided in the supplementary materials. Specifically, we systematically searched the scientific databases PubMed/MEDLINE, SPORTDiscus, and Scopus on 17 September 2024, to identify all relevant studies according to our PICO framework. We identified all English-language, peer-reviewed research studies that induced any form of global muscle hypertrophy (i.e., hypertrophy induced by resistance exercise, drugs, or a combination of treatments) in humans, animals, and transgenic animal models. In addition, the studies had to report changes in glucose metabolism (i.e., HbA1c or blood glucose) or fat mass. In our analysis, we included 122 studies [22, 49–169] that included 99 human and 23 animal interventions studies (see Online Supplementary Appendix 4 and 5 for details).

### Is Muscle Hypertrophy Associated with Altered Fat Mass?

**Fig. 2:**
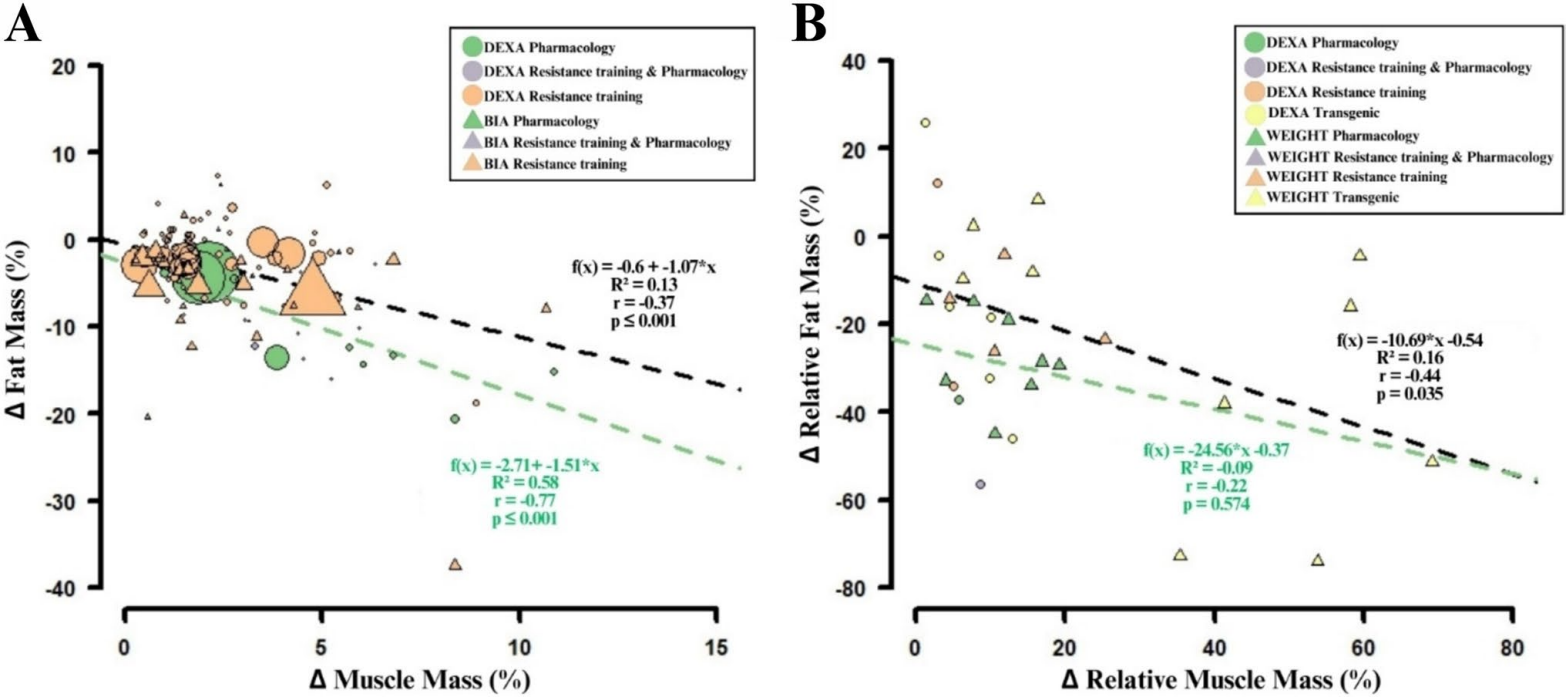
Scatter plots of the effect of muscle hypertrophy on fat mass changes. **A** represents changes in muscle mass/fat mass from pre- to post-intervention in humans. The green dashed line indicates the regression line of the pharmaceutical treatment (*n* = 15, *k* = 21), while the black dashed line includes all other treatments (*n* = 82, *k* = 109). The size of each data point represents the sample size. **B** represents relative differences in muscle mass versus relative differences in fat mass between experimental group and controls in animal models. The green dashed line indicates the regression line of the pharmaceutical treatment (*n* = 5, *k* = 9), while the black dashed line includes all other treatments (*n* = 16, *k* = 23)

To find out whether muscle hypertrophy alters fat mass, we analyzed 116 studies (Human interventions: *n* (included studies) = 95, 2702 participants, *k* (included outcomes) = 130; animal models: *n* = 20, *k* = 32; Fig. 2). Muscle and fat mass were primarily assessed using dual-energy X-ray absorptiometry (DEXA) or bioelectrical impedance analysis (BIA). In these studies, skeletal muscle hypertrophy was induced by drugs (DEXA: *k* = 21; BIA: *k* = 0), resistance training (DEXA: *k* = 75; BIA: *k* = 31), or a combination of both (DEXA: *k* = 3; BIA: *k* = 0).

To isolate the effects of pharmaceutical interventions, we first examined studies without exercise components (*k* = 21). These interventions resulted in an average increase in muscle mass of 4.1 ± 2.6% and a reduction in fat mass of 8.9 ± 5.0%, which was always measured with DEXA. There was a significant, inverse correlation between muscle hypertrophy and fat mass reduction (*R*^2^ = 0.58, *r* = − 0.77, *p* < 0.001; Fig. 2A). We then analyzed resistance training trials (DEXA: *k* = 75; BIA: *k* = 31), which showed an average increase in muscle mass of 2.3 ± 1.9% and a reduction in fat mass of 3.0 ± 5.4%. A small number of studies (*k* = 3; all DEXA) combined resistance training with pharmaceutical agents. These studies reported the greatest improvements, showing an increase in muscle mass of 4.7 ± 1.5% and a reduction in fat mass of 9.6 ± 5.9%. Across all human interventions, muscle mass increased by an average of 2.7 ± 2.1%, accompanied by a fat mass reduction of 4.1 ± 5.8%. Overall, muscle hypertrophy was significantly associated with fat loss (*R*^2^ = 0.25, *r* = − 0.51, *p* < 0.001).

This trend held true across subgroups stratified by body mass index (BMI; normal: *R*^2^ = 0.49, *p* < 0.001; overweight: *R*^2^ = 0.21, *p* < 0.001; obese: *R*^2^ = 0.26, *p* < 0.05), age (young: *R*^2^ = 0.18, *p* < 0.05; middle-aged: *R*^2^ = 0.40, *p* < 0.001; elderly: *R*^2^ = 0.50, *p* < 0.001), and training experience (inexperienced: *R*^2^ = 0.38, *p* < 0.001; experienced: *R*^2^ = 0.20, *p* < 0.05; see Supplementary Appendix 6).

In animal studies, researchers measured muscle mass by DEXA or by dissecting and weighing muscle tissue (e.g., hindlimb muscles; WEIGHT). Muscle hypertrophy was stimulated either by drugs (WEIGHT: *n* = 4, *k* = 8 including rats [*k* = 5], and mice [*k* = 3]; DEXA: *n* = 1, *k* = 1 including rats only), animal resistance training/muscle loading (WEIGHT: *n* = 4, *k* = 4 including rats [*k* = 2], and mice [*k* = 2]; DEXA: n = *2*, *k* = 2 including rats only), a combination of both (WEIGHT: *n* = 0, *k* = 0; DEXA: *n* = 1, *k* = 1 including rats only), or genetic manipulation (WEIGHT: *n* = 8, *k* = 10 including mice [*k* = 9] and pigs [*k* = 1]; DEXA: *n* = 2, *k* = 6 including mice only).

Pharmaceutical-only interventions (*k* = 9, including rats [*k* = 6] and mice [*k* = 3]) increased relative muscle mass by 10.4 ± 5.8% and reduced relative fat mass by 28.4 ± 9.8% when compared with untreated control animals. However, the linear regression between muscle mass increase and fat mass decrease was not significant for pharmaceutical-only interventions (*R*^2^ = − 0.09, *r* = − 0.22, *p* = 0.574; Fig. 2B). Resistance training-only interventions (*k* = 6 including rats [*k* = 4] and mice [*k* = 2]) had greater muscle mass (10.0 ± 7.6%) and less fat mass (15.1 ± 15.3%) than their controls. The combination of resistance training and pharmaceutical intervention was only used by Cavalie et al. [66] in rats and resulted in a relative increase in muscle mass of 9% and a relative decrease in fat mass of 56% compared with controls. Transgenic modification (*k* = 16 including mice [*k* = 15] and pigs [*k* = 1]) resulted in 25.3 ± 22.8% more muscle mass and 22.2 ± 27.3% less fat mass than in wildtype controls. When data from resistance training interventions, data from resistance training combined with pharmaceutical administration, and transgenic models are combined, there is a significant relationship (*R*^2^ = 0.16, *r* = − 0.44, *p* = 0.035; Fig. 2B). Combining all animal data, muscle hypertrophy stimulation increased muscle mass by 17.7 ± 18.4% and reduced fat mass by 23.7 ± 22.3% compared with controls. This relationship was significant (*R*^2^ = 0.12, *r* = − 0.38, *p* = 0.031). The use of absolute muscle mass values (*n* = 15, mice: *n* = 9, pigs: *n* = 2, rats: *n* = 4, *k* = 24; mice: *k* = 11, pigs: *k* = 2, rats: *k* = 11) also yielded significant results (*R*^2^ = 0.17, *r* = − 0.46, *p* = 0.025).

In summary, this systematic search and quantitative analysis of human and mouse studies confirms that skeletal muscle hypertrophy is associated with reduced fat mass.

### Is Muscle Hypertrophy Associated with Altered Glucose Homeostasis?

**Fig. 3:**
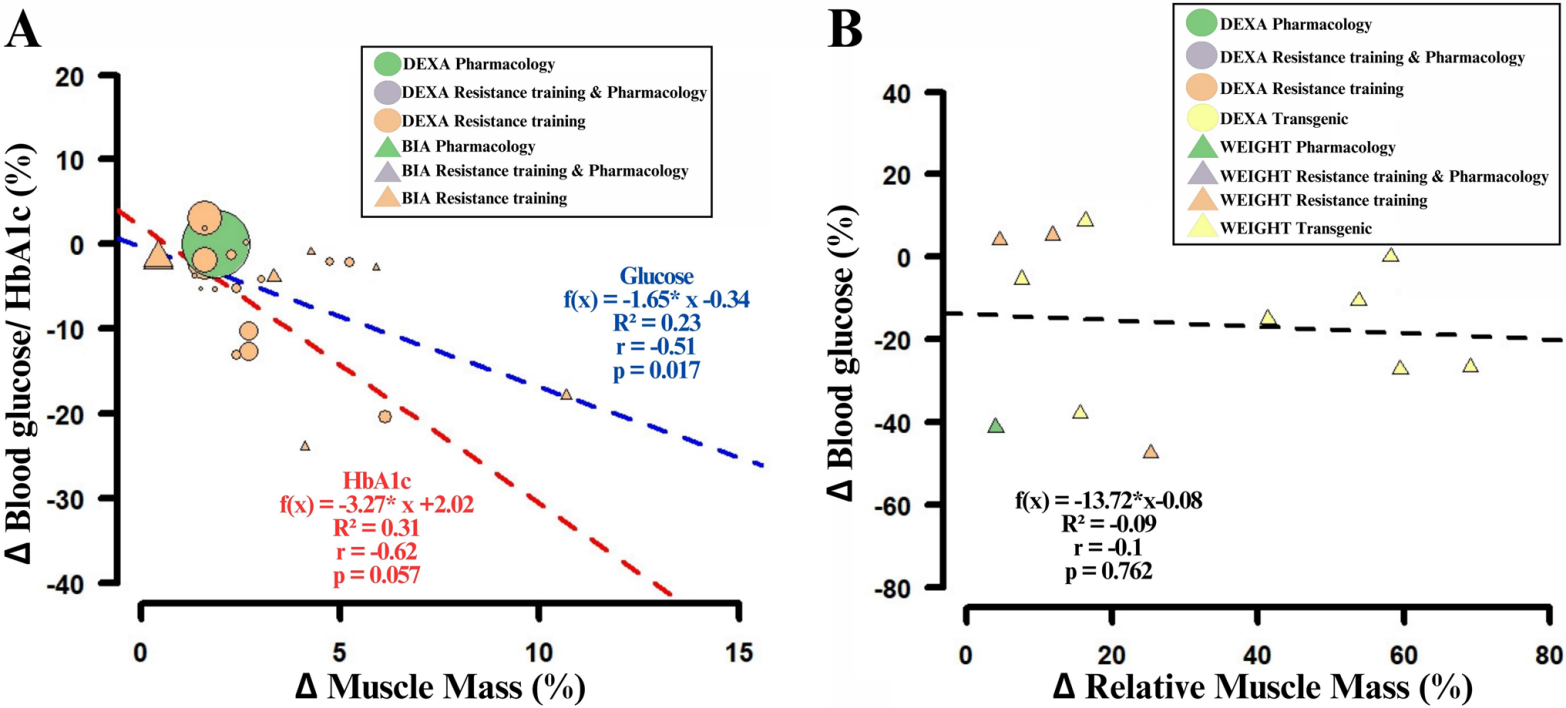
Scatter plots of the effect of muscle hypertrophy on glucose homeostasis. The dashed lines indicate the linear regression. **A** represents changes in muscle mass and fasted glucose or HbA1c concentrations from pre- to post-intervention in humans (*n* = 22, *k* = 31). The size of each data point represents the sample size. **B** represents relative differences in muscle mass versus differences in blood glucose concentration between experimental groups and controls in animal models (*n* = 10, *k* = 12)

Our literature search yielded 32 studies (human *n* = 22, *k* = 31, 553 participants; animals *n* = 10, *k* = 12; Fig. 3). To minimise confounding effects from exercise, we first aimed to analyse pharmaceutical interventions without exercise. However, only a single human study [75] met this criterion. It reported an increase in muscle mass of 1.87% following the administration of testosterone, with no change in fasting blood glucose levels, which remained at 5.6 mmol/L. In contrast, resistance training (*k* = 30) increased muscle mass by 1.9 ± 0.9% and reduced the HbA1c by 4.1 ± 4.8% (*k* = 10). In studies that reported fasting glucose, muscle mass increased by 3.4 ± 2.3%, and glucose levels declined by 6.1 ± 7.3% (*k* = 20). No studies combining resistance training and pharmaceutical treatment reported blood glucose. Owing to the near-complete absence of pharmaceutical-only human studies, no regression analyses were performed for this group.

When pooling available data, the pooled interventions increased muscle mass in humans by 1.9 ± 0.9%, while HbA1c decreased by 4.1 ± 4.6% (relative change). In studies where fasting blood glucose was the primary endpoint, muscle mass increased by 3.3 ± 2.3% and glucose levels decreased by 5.8 ± 7.3%. The linear regression for HbA1c revealed a nonsignificant but suggestive trend toward an inverse association with muscle hypertrophy (*R*^2^ = 0.31, *r* = − 0.62, *p* = 0.057). However, fasting glucose showed a statistically significant inverse relationship with muscle gain (*R*^2^ = 0.23, *r* = − 0.51, *p* = 0.017; Fig. 3A).

We also observed a trend suggesting a greater improvement of glucose homoeostasis in individuals classified as overweight or obese on the basis of BMI (normal: 20–24.9 kg/m^2^, *R*^2^ = 0.24, *p* = 0.672; overweight: 25–29.9 kg/m^2^, *R*^2^ = 0.18, *p* = 0.129; obese: ≥ 30 kg/m^2^, *R*^2^ = 0.46, *p* < 0.05) and in different age groups (young: 18 ≤ 39 years, *R*^2^ = 0.55, *p* < 0.05; middle age: 40 ≤ 64 years, *R*^2^ = 0.51, *p* < 0.05; elderly: ≥ 65 years, *R*^2^ = 0.65, *p* < 0.05; see Online Supplementary Appendix 7). No studies were identified that reported glucose outcomes in resistance-experienced individuals.

In animal studies, glucose regulation was primarily assessed via fasted or non-fasted blood glucose levels (*n* = 10, *k* = 12). HbA1c was reported in only one study involving myostatin knockout mice, which demonstrated a relative increase in muscle mass of 53.8–59.5% and a corresponding HbA1c reduction of 2.2–21.8% [61]. Owing to the limited availability of HbA1c data, subsequent analyses focused exclusively on blood glucose concentrations. Animal models inducing hypertrophy and reporting glucose outcomes included pharmaceutical interventions (*n* = 1, *k* = 1, mice), resistance training (*n* = 3, *k* = 3; rats [*k* = 2], mice [*k* = 1]), and transgenic modification (*n* = 6, *k* = 8; mice [*k* = 7], pigs [*k* = 1]).

In the only pharmaceutical study [119], mice exhibited a 4% increase in muscle mass and a 41% reduction in blood glucose compared with controls. No regression analysis was performed owing to the single dataset. Resistance training models (*k* = 3; rats [*k* = 2], mice [*k* = 1]) reported a 13.9 ± 8.6% increase in muscle mass and a 12.7 ± 24.6% reduction in blood glucose compared with controls. None of the included studies that combined resistance training with pharmaceutical treatment evaluated glucose parameters.

Transgenic modification models (*k* = 8; mice [*k* = 7], pigs [*k* = 1]) showed a 40.2 ± 22.2% higher muscle mass and a 14.3 ± 14.6% lower glucose concentration compared with controls. When all animal data were combined, muscle mass in the treatment groups was on average 30.6 ± 23.2% higher, while blood glucose levels were 16.1 ± 18.7% lower than in controls. However, linear regression analysis did not reveal a statistically significant relationship (*R*^2^ = − 0.09, *r* = − 0.10, *p* = 0.762; Fig. 3B). The same was true for analyses using absolute muscle mass values (*n* = 11, *k* = 13; mice [*k* = 10], pigs [*k* = 1], rats [*k* = 2]), where no significant correlation was found (*R*^2^ = − 0.057, *r* = 0.176, *p* = 0.566).

In summary, resistance training is associated with improved glucose homeostasis in humans. However, the independent contribution of muscle hypertrophy to these effects remains uncertain and warrants further investigation. In contrast, experimental animal models more consistently support a link between increased muscle mass and reduced blood glucose levels.

## Potential Mechanisms Underlying the Effects of Muscle Hypertrophy on Fat Mass and Glucose Homeostasis

**Fig. 4:**
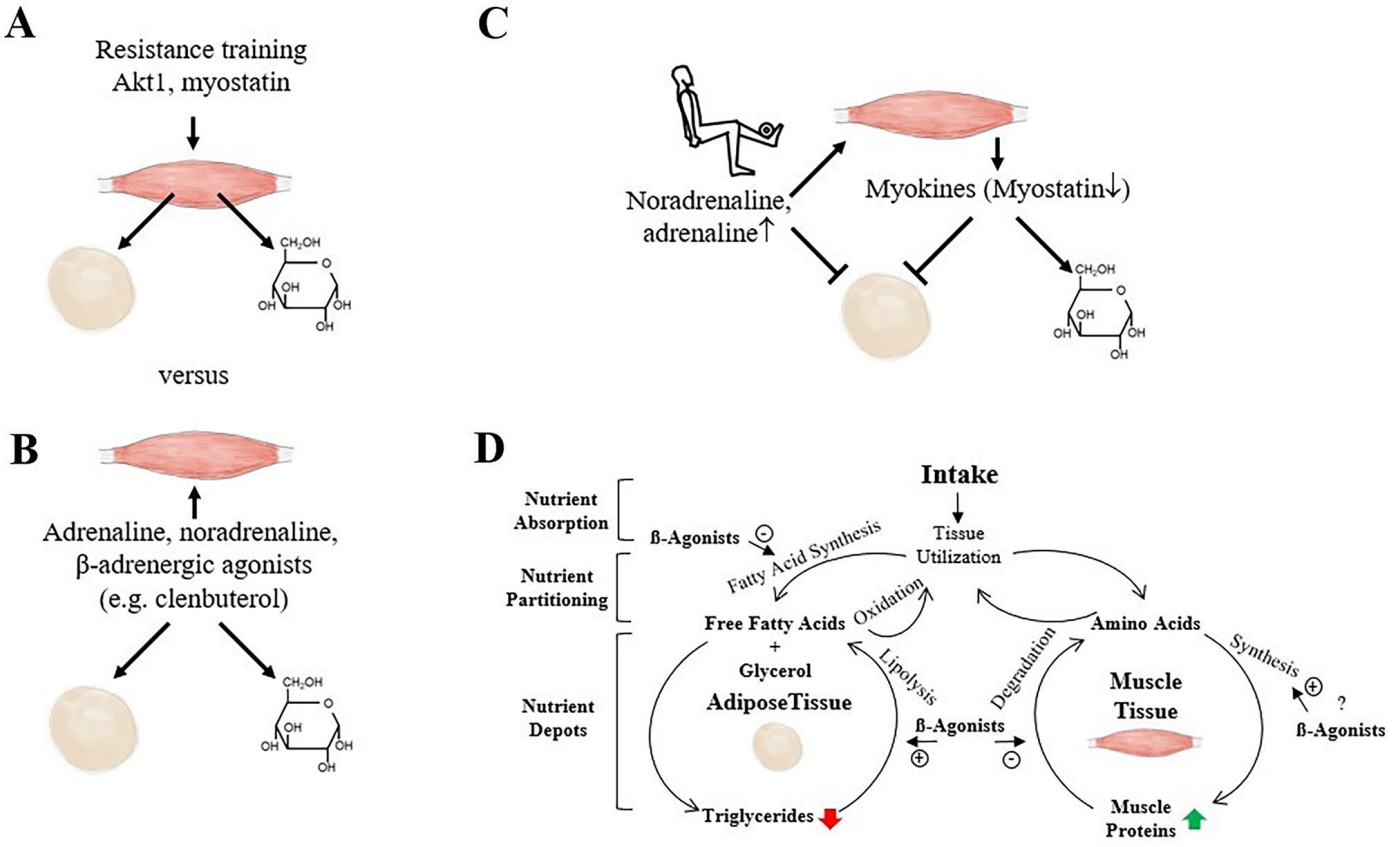
Mechanisms that explain the effects of muscle hypertrophy on fat mass and glucose homeostasis. **A** Akt1 and myostatin are examples of regulators whose action on muscle is enough to cause a loss of fat mass and improvement of glucose homeostasis. **B** In contrast, the catecholamines adrenaline and noradrenaline, as well β-adrenergic agonists, act both on skeletal muscle and adipose tissue as both tissues express adrenergic receptors. **C** Inter-organ signal transduction mechanisms (e.g., in response to resistance exercise) versus **D** metabolic mechanisms (e.g., β-adrenergic agonist effects on skeletal muscle and adipose tissue metabolism; [170]) that can potentially explain the effects of muscle hypertrophy on fat and glucose

The systematic analysis of the literature supports our conclusion from subjectively chosen publications that muscle hypertrophy is associated with fat loss and improved glucose homeostasis in animals and humans. In relation to this, we ask two questions that are illustrated in Fig. 4:

1. Are the blood glucose and fat mass lowering effects of muscle hypertrophy-inducing interventions a consequence of muscle hypertrophy (i.e., it is sufficient to induce muscle hypertrophy to trigger fat loss and an improved glucose homeostasis as secondary effects; Fig. 4A) or do the interventions target muscle, adipose tissue, and glucose metabolism in parallel (Fig. 4B)?
2. How much of the muscle, fat, and glucose homeostasis effects can be explained by signal transduction, e.g., through myokines (Fig. 4C) and how much is explained by metabolic mechanisms such as a “metabolite steal” by hypertrophying skeletal muscle (Fig. 4D)?

Before answering these questions, we will briefly review the regulation of skeletal muscle hypertrophy and glucose uptake.

### Regulation of Skeletal Muscle Hypertrophy and Glucose Uptake

The signal transduction pathways that regulate skeletal muscle glucose uptake and muscle hypertrophy overlap. At the center is the AKT serine/threonine kinase–mammalian/mechanistic target of rapamycin (AKT–mTOR) pathway. However, transgenic mouse studies and other experiments demonstrate that other kinases, phosphatases and signaling molecules are also involved in regulating glucose uptake and muscle hypertrophy. Here, we will first contrast the regulation of insulin-stimulated skeletal muscle glucose uptake with the IGF-1, mechanical load (i.e., the stimulus induced by resistance training), or leucine-stimulation of protein synthesis and muscle hypertrophy. After that we will discuss other signaling proteins that regulate both glucose uptake and protein synthesis in muscle.

Insulin-stimulated glucose uptake and glycogen synthesis has been extensively reviewed [171]. It starts with insulin binding to the insulin receptor (encoded by *INSR*). This causes the insulin receptor to tyrosine-autophosphorylate itself, which in turn recruits the insulin receptor substrate (IRS) to the insulin receptor. This event then activates the serine/threonine kinase AKT (note there are three AKT isoforms, abbreviated as AKT1–3) via phosphoinositide 3-kinase (PI3K) and 3-phosphoinositide-dependent kinase 1 (PDK1) as intermediary kinases. Active AKT is then capable of phosphorylating several substrates. One of them is AKT substrate of 160 kDa (AS160) which is the key AKT substrate for increasing glucose uptake. Specifically, AKT-phosphorylated AS160 causes intracellular vesicles that contain glucose transporter 4 (GLUT4) to move and fuse with the plasma membrane of the muscle fiber, termed sarcolemma. As a consequence, the increased number of GLUT4s in the sarcolemma will increase glucose uptake. AKT also activates glycogen synthase via intermediate steps, and this increases glycogen synthesis from the glucose that has entered the muscle fiber. However, skeletal muscle glucose uptake is not only activated by insulin, but also by exercise, via mechanisms that are independent of insulin [171, 172].

Insulin-like growth factor-1 (IGF-1) is a growth factor similar to insulin, but in contrast to insulin, IGF-1 primarily stimulates muscle protein synthesis and muscle hypertrophy. Circulating IGF-1 binds to the IGF-1 receptor (encoded by IGF1R). While both insulin and IGF-1 activate AKT, IGF-1-stimulated AKT primarily activates the serine/threonine protein kinase mTOR. mTOR, in turn, interacts with the tuberous sclerosis complex (TSC), particularly TSC1. mTOR exists as part of two distinct multi-protein complexes: mTOR complex 1 (mTORC1) and mTOR complex 2 (mTORC2). Of these two complexes, active mTORC1 increases muscle protein synthesis and hypertrophy. However, IGF-1 is not the only activator of mTORC1, as mTORC1 is also activated by mechanical stimuli, e.g., induced through resistance training [173], and by leucine, which binds to the leucine sensors sestrin 1/2 [174, 175]. If mTORC1 is activated by one of these stimuli, it increases muscle protein synthesis, which involves ribosomes translating mRNA into protein. The ribosome is an organelle that synthesizes proteins based on the amino acid sequences encoded in the protein-coding mRNAs that bind to the ribosome. Specifically, activation of mTORC1 promotes the initiation and elongation of the translation of mRNA into protein. However, mTORC1 increases not only protein synthesis from existing ribosomes but also the synthesis of more ribosomes, a process known as ribosomal biogenesis [176].

While the insulin/IGF-1–AKT1–mTORC1 pathway is central, many other regulatory proteins additionally regulate glucose uptake and muscle hypertrophy, with many of these proteins regulating both. To identify genes and proteins that contribute to glucose uptake and muscle hypertrophy, we have systematically searched the literature for genes whose transgenesis increases glucose uptake [177] or causes skeletal muscle hypertrophy [32]. We found that the transgenesis of *Mstn*, *Foxc2*, *Fst*, *Ccn5*, *Cpt1b*, *Hdac3*, *Insr*, *Steap4*, *Igfbp3*, *Tnfsf11*, *Inpp5k*, and *Nos2* affects both skeletal muscle glucose uptake and promotes skeletal muscle hypertrophy in mice, confirming an overlap of the underlying regulatory mechanisms. The most prominent among these genes is *Mstn,* which encodes myostatin, an inhibitor of muscle growth. The loss of this gene causes muscle hypertrophy, or double muscling, in many species, including mice [38] and humans [39]. If *Mstn* is knocked out in a diabetic, overweight mouse strain, then this not only results in muscle hypertrophy but additionally causes profound fat loss and improves glucose homeostasis after a glucose challenge [21]. Increased glucose uptake by growing tissues is potentially explained by the fact that glucose is not only a fuel but also a building block, as e.g., RNA and DNA are made from nucleotides that combine a sugar, typically derived from glucose, a base, and a phosphate. Thus, if a hypertrophying muscle fiber synthesizes more ribosomes, which are mainly made from ribosomal RNA, it will convert more glucose into nucleotides to synthesize that additional ribosomal RNA. Furthermore, we and others have found that the ^14^C from ^14^C glucose ends up in protein, and that stimulating protein synthesis increases the rate at which this occurs [178]. This incorporation of ^14^C from ^14^C glucose into protein can only be explained by a glucose → glycolytic intermediates + NH_2_ donor → non-essential amino acids → protein pathway. This should not come as a surprise, given that almost 100 years ago, Otto Warburg demonstrated that sarcomas, which are a growing tissue like hypertrophying muscle take up glucose at a high rate [179]. Moreover, the group of the cancer metabolism researcher Matt Vander Heiden has demonstrated that 20–30% of glucose-derived ^14^C ends up in protein and a similar amount of ^14^C in RNA and DNA in cultured H1299, A549, and A172 cancer cells [180]. This again demonstrates that glucose is not just a fuel but also a building block, as it is a substrate for several anabolic reactions that are active in growing tissues.

### Causality or Parallelism? Disentangling the Effects of Muscle Hypertrophy on Fat Mass and Glucose Homeostasis

The answer to the first question is that the stimulation of skeletal muscle hypertrophy is sufficient to lower fat mass and improve glucose homeostasis as secondary effect. However, some repartitioning treatments such as β-adrenergic agonists act on skeletal muscle and adipose tissue at the same time.

The sufficiency of skeletal muscle hypertrophy for fat loss has been demonstrated using mouse models in which the transgene that induces hypertrophy only targets skeletal muscle. For example, the stimulation of skeletal muscle hypertrophy by expressing constitutively active Akt1 [22] only within muscle, or by knocking down the myostatin (i.e., activin IIB) receptor only in muscle [181], demonstrates that muscle hypertrophy is sufficient to cause fat loss and improve glucose homeostasis as secondary effects. Evidence that myostatin needs to act on skeletal muscle to cause fat loss comes from a study where the knock out myostatin receptor in adipose tissue had no effect on body composition, weight gain, or glucose and insulin tolerance in mice fed a standard or high-fat diet [181]. These experimental findings are mirrored by pharmaceutical interventions that act primarily on skeletal muscle. Bimagrumab, a monoclonal antibody that blocks the activin type IIB receptor, has shown promising results in humans. In a 48-week randomized trial involving individuals with type 2 diabetes and BMIs between 28–40 kg/m^2^, bimagrumab increased lean mass by 1.70 kg (80% CI: 1.14 to 2.26 kg), reduced fat mass by 7.49 kg (80% CI: − 8.33 to − 6.64 kg), and decreased HbA1c by 0.76% (80% CI: − 1.05 to − 0.48%) [23]. A recent systematic review and meta-analysis confirmed these dual effects of bimagrumab on increasing muscle mass and reducing fat mass in humans [182].

However, some treatments clearly act on skeletal muscle and adipose tissue at the same time. A key example is β-adrenergic agonists, which stimulate skeletal muscle hypertrophy and adipose lipolysis by acting on adrenergic receptors expressed by both tissues [170, 183]. Another example is testosterone, which binds to androgen receptors in skeletal muscle, activating anabolic pathways such as mTORC1 and promoting protein synthesis [184, 185]. In addition, testosterone exerts direct effects on adipose tissue by inhibiting the adipogenic differentiation of mesenchymal stem cells, reducing lipid accumulation, and stimulating lipolysis through androgen receptor-mediated signalling [186, 187]. In summary, the stimulation of skeletal muscle hypertrophy suffices to cause a loss of fat mass and improve glucose homeostasis in organisms where it is deranged. However, some treatments, such as β2 agonists and testosterone, act on both skeletal muscle and adipose tissue simultaneously.

### Signal Transduction Versus Metabolic Mechanisms in Muscle–Fat–Glucose Crosstalk

The second question is about the importance of inter-organ signaling versus metabolic mechanisms. Is it that the effect of skeletal muscle hypertrophy on adipose tissue mass is primarily explained by signaling molecules such as myokines? Or is it that hypertrophying muscles “steal” molecules from the blood, reducing the availability of these molecules for adipose tissue, and causing it to shrink? In terms of signaling, we will only discuss inter-organ signaling. As with all forms of exercise, the concentrations of the catecholamines noradrenaline (American English, “norepinephrine”) and adrenaline (American English, “epinephrine”) increase in blood during resistance training [188]. The increase in noradrenaline and adrenaline following resistance exercise is clearly not the primary stimulus for muscle hypertrophy; otherwise, training a single muscle would be sufficient to cause whole-body muscle hypertrophy. However, the rise in catecholamines during resistance training [188] should stimulate white adipose tissue lipolysis [183] and activate brown adipose tissue heat production [189], which will increase energy expenditure. Resistance exercised and hypertrophying muscles will also alter the release of myokines, i.e., hormone-like regulators, such as myostatin [190]. After resistance exercise, myostatin expression significantly decreases in human skeletal muscle (meta-analysis of human biopsy studies, [191]). A decrease of circulating myostatin can explain fat loss and improved glucose homeostasis, as this is a phenotype of *Mstn*^−/−^ mice [21]. As mentioned above, this effect is dependent on the action of myostatin on skeletal muscle, as knock out of the myostatin receptor in skeletal muscle but not in fat causes fat loss and improved glucose homeostasis [181]. In summary, skeletal muscle hypertrophy induced by resistance training or other measures will cause a release of hormones from glands or skeletal muscle that act on white adipose tissue and glucose metabolism. Such circulating regulators can at least partially explain how skeletal muscle can affect fat mass and glucose homeostasis.

A second way to view the effects of muscle hypertrophy on fat mass and glucose homeostasis is to view them from a metabolic perspective. In relation to this we will discuss the idea that a hypertrophying muscle “steals” metabolites from other tissues. This idea was formulated by the group of Alexandra McPherron, who found that myostatin knock out-induced muscle hypertrophy results in fat loss and improved glucose homeostasis in mice [181]. Hypertrophying muscles must take up 1 g of metabolites from the circulation for each 1 g of dry biomass that they build. Thus, if muscle is 70% water and 30% dry mass, the generation of 1 kg of new muscle implies that 300 g of metabolites must be taken up or are “stolen” from the circulation. Since dry muscle mass is largely made up of protein (≈ 70%), it contains significant amounts of amino acids, particularly branched-chain amino acids such as leucine, isoleucine, and valine. Elevated circulating branched-chain amino acids are an established risk marker for the development of type 2 diabetes mellitus [192]. Thus, the increased uptake of branched-chain amino acids may contribute to improved glucose homeostasis. However, this metabolite “steal” by hypertrophying muscles only functions during active hypertrophy and not in organisms where the muscles are in a hypertrophied steady state, such as a fully grown adult myostatin knockout animal.

## Implications for Clinical Practice

The results of our systematic literature review suggest that the stimulation of skeletal muscle hypertrophy is sufficient to cause anti-obesity and anti-diabetic effects. Having another therapy for metabolic diseases is desirable because the high number of patients that are overweight, obese, and diabetic worldwide suggests that lifestyle interventions and existing treatments are not effective enough for a large enough proportion of people.

To contextualise the clinical relevance of our findings, it is important to consider the magnitude of the observed changes in metabolic parameters relative to established diagnostic thresholds and therapeutic goals. Across the included studies, skeletal muscle hypertrophy was associated with a mean relative reduction in fat mass of − 4.1 ± 5.8% (range − 37.5% to 7.3%), HbA1c of − 4.1 ± 4.6% (range − 12.6% to 0%), and fasting glucose of − 5.8 ± 7.3% (range − 23.9% to 3.1%). Assuming a baseline HbA1c of 8.0%—a common value in individuals with poorly controlled type 2 diabetes—this corresponds to an absolute reduction of approximately 0.3%, lowering HbA1c to around 7.7%. While a reduction of ≥ 0.5% is typically considered clinically meaningful and associated with a lower risk of diabetes-related complications [193, 194], our findings still indicate a favorable trend in glycemic control. Likewise, a mean 5.8% reduction in fasting glucose, for example from 130 mg/dL to approximately 122.5 mg/dL, is clinically relevant. This difference spans the diagnostic boundary between diabetes mellitus (≥ 126 mg/dL) and prediabetes (< 126 mg/dL) [195]. Regarding adiposity, current clinical guidelines recommend a reduction in total body weight of approximately 3–7% to achieve improvements in metabolic parameters, including glycemic control and cardiovascular risk [196–198]. The mean fat mass reduction observed in our review (− 4.1%) closely aligns with these thresholds, and importantly, reflects a direct change in adiposity rather than general body weight—thereby reinforcing the potential clinical value of muscle hypertrophy as a mechanism for improving body composition.

By contrast, a recent meta-analysis [199] focused exclusively on whether resistance training–induced muscle hypertrophy enhances acute insulin-mediated glucose uptake, as measured by oral glucose tolerance test (OGTT), euglycemic–hyperinsulinemic clamp, or intravenous glucose tolerance test (IVGTT). Although the analysis confirmed increases in both fat-free mass and short-term glucose disposal, it found no consistent correlation between the two. By comparison, we evaluated longer-term, steady-state markers—HbA1c and fasting glucose—which reflect chronic glycemic control rather than transient responses to a glucose load or insulin infusion. Our findings demonstrate an association between increased muscle mass and improved HbA1c, fasting glucose, and fat mass, suggesting that hypertrophy supports sustained metabolic benefits (e.g., improved baseline glucose homeostasis), even if it does not necessarily boost the rate of acute glucose clearance.

Several obesity and diabetes mellitus associations and position statements already recommend resistance training as an intervention [200–202]. They base this on the fact that resistance training prevents the loss of lean/muscle mass that normally occurs if people with obesity start an energy-restricted/hypocaloric diet [203] and that resistance training improves glucose homeostasis (e.g., [204]). However, to our knowledge, none of these associations and position statements argue that muscle hypertrophy is an outcome that patients should aim for, as the relationship between muscle hypertrophy, fat mass, and glucose homeostasis is not discussed. Also, none of the associations and position statements recommend skeletal muscle hypertrophy-inducing drugs, such as testosterone, androgenic/anabolic steroids, β-adrenergic agonists, or emerging drugs that target, e.g., the myostatin pathway, such as bimagrumab [23]. Likely reasons are that the anti-obesity and anti-diabetic effects of muscle hypertrophy are underappreciated, that these drugs have received bad publicity as doping agents, and that some of these muscle-building drugs have side effects. For example, potential side effects of testosterone and anabolic, androgenic steroids usage can be cardiovascular disease, liver damage, testicular atrophy, breast development in men (gynecomastia), and kidney damage [43, 205]. Side effects of β-adrenergic agonists are tachycardia, tremors, palpitations, nervousness, headaches, and hyperglycaemia [205]. However, such side effects are dose-dependent and do not occur in all users.

Given that the anti-obesity and anti-diabetic effects of muscle hypertrophy and of muscle-building drugs are underappreciated, it makes sense to us to start clinical trials to re-evaluate the cost–benefit ratio of these drugs in patients who are obese and those with diabetes mellitus. Moreover, given that the effects of these drugs and resistance training are additive [44], we suggest trials in patients with obesity or type 2 diabetes mellitus where hypertrophy-focused resistance training and moderate doses of anabolic, androgenic steroids and/or β-adrenergic agonists are combined. Moreover, the drug treatment regime could be personalised, e.g., by basing it on free testosterone levels or the absence of side effects. Also, there is a lot of unscientific information on muscle-building drugs that is based on the widespread use of such drugs (e.g., 1% of the population are estimated to use anabolic steroids) over decades (www.steroids.com [206]). However, the most recent estimate reports a lifetime prevalence of ever using anabolic androgenic steroids among males in the general population of 1–5%, which is much more common in males than in females (> 50:1) [207]. To us, it makes sense to study this misinformation and to test clinically relevant claims and treatment strategies in clinical trials.

Recently the bimagrumab trial in patients with obesity and type 2 diabetes mellites has demonstrated that not only testosterone, anabolic, androgenic steroids, and/or β-adrenergic agonists have anti-obesity and anti-diabetic effects but that the myostatin pathway can also be effectively targeted in humans to lose fat and to improve glucose homeostasis [23]. Versanis, the company that holds the rights for bimagrumab was bought by Lilly in 2023 [208], suggesting that targeting muscle hypertrophy as a means to treat obesity and type 2 diabetes mellitus is now appreciated by major pharmaceutical companies. In an opinion in the *Journal of Physiology* [209], we have recently argued that the bimagrumab effects in patients with obesity are preferable to those of, e.g., semaglutide because the fat loss is not associated with muscle atrophy, as is the case with semaglutide treatment [210]. Moreover, at this early stage it seems that bimagrumab has fewer side effects than testosterone, anabolic, androgenic steroids, and/or β-adrenergic agonists. A key reason for this is that bimagrumab and myostatin act primarily on skeletal muscle, whereas the androgen receptor and adrenergic receptors are expressed by many organs.

Beyond the clinical and mechanistic implications of our findings, there are important considerations regarding the variables for future interventional studies. Given the growing interest in pharmaceutical agents that promote skeletal muscle hypertrophy (e.g., myostatin inhibitors, selective androgen receptor modulators [SARMs], or β2-adrenergic agonists), future trials may increasingly be driven by pharmaceutical industry stakeholders seeking to demonstrate additive or superior value compared with non-commercial lifestyle interventions. Such trends have already been observed in the context of obesity and diabetes pharmacotherapy [211]. While academic institutions will remain essential for early-stage mechanistic research and the testing of combined approaches (such as drug and exercise interventions), large-scale efficacy and cost-effectiveness trials are likely to be increasingly supported by the pharmaceutical industry owing to the commercial potential of these interventions [212]. Nevertheless, it is crucial to carefully consider the translational relevance of these findings to real-world populations. Exercise adherence is notoriously low among free-living individuals with obesity or type 2 diabetes, particularly when interventions are unsupervised or not integrated into clinical support systems [213–215].

In summary, while resistance training is recommended for patients with obesity and diabetes mellitus, it seems that the anti-obesity and anti-diabetic effects of muscle hypertrophy per se are underappreciated by those that treat patients with obesity and diabetes mellitus. For this reason, and to increase the options for treatment, we recommend three things:

1. Start programs to develop next generation drugs and interventions that stimulate global muscle hypertrophy. There are plenty of targets, as a systematic review of genes whose gain or loss-of-function causes muscle hypertrophy has identified 47 genes in 2018 [32]. A key aim should be to develop drugs and interventions that have fewer side effects than testosterone, anabolic, androgenic steroids, and/or β-adrenergic agonists. Bimagrumab is arguably the first of such next generation drugs.
2. Taking in mind the anti-obesity and anti-diabetic effects of resistance training, testosterone, anabolic, androgenic steroids, β-adrenergic agonists, and bimagrumab, re-evaluating the cost–benefit relationship of these drugs alone and in combination with hypertrophy-focused resistance training for patients who do not mind becoming more muscular. Also, studying the unscientific information from users of muscle-building drugs could be useful to then test disease-relevant claims in clinical trials.
3. The body mass index (BMI) is a poor indicator for evaluating the success of weight loss interventions as it does not inform whether muscle/lean or fat mass is lost. All weight loss studies should report both changes of muscle/lean mass and of fat mass. This can be achieved by BIA and DEXA measurements.

## Summary and Conclusions

Muscle hypertrophy has clinically meaningful anti-diabetic and anti-obesity effects in both humans and animals. Importantly, the stimulation of muscle hypertrophy is sufficient to trigger fat loss and, particularly in animal models, improvements in glucose homeostasis as secondary effects. In humans, resistance training improves glucose metabolism, but the specific role of muscle hypertrophy in this process remains uncertain and warrants further investigation. Some muscle hypertrophy-inducing treatments such as β-adrenergic agonists not only target muscle but also white adipose tissue lipolysis and brown fat thermogenesis. Hypertrophying muscles can affect the function and metabolism of other tissues via myokines or a steal of metabolites, as hypertrophying muscles must take up more metabolites to build new biomass. The observation that muscle hypertrophy also triggers fat loss and improves glucose homeostasis should lead to clinical trials that investigate the effect of muscle hypertrophy stimulation through resistance training alone or in combination with drugs on metabolic outcomes, especially in elderly patients with metabolic disease. In these trials it is important to not just report body weight but to quantify the changes of muscle or lean mass and fat mass separately.

## Supplementary Information

Below is the link to the electronic supplementary material.

Supplementary file1 (PDF 392 KB)

Supplementary file2 (XLSX 126 KB)

Supplementary file3 (XLSX 124 KB)

## Declarations

### Availability of Data and Material

All data and material can be found in the supplementary material.
