# Supplementary material for: Effects of Skeletal Muscle Hypertrophy on Fat Mass and Glucose Homeostasis in Humans and Animals: A Narrative Review with Systematic Literature Search
Source: Sports Med. 2025 Jun 27;55(8):1867–85. doi: 10.1007/s40279-025-02263-w (PMC12460530; doi:10.1007/s40279-025-02263-w)
Supplement: Supplementary file 1 — Supplementary file1 (PDF 392 KB) [file 40279_2025_2263_MOESM1_ESM.pdf]

# Effects of skeletal muscle hypertrophy on fat mass and glucose homeostasis in humans and animals – a narrative review with systematic literature search

Tim Havers*, Steffen Held, Martin Schönfelder, Stephan Geisler †, Henning Wackerhage†

[1] Department of Fitness and Health, IST-University of Applied Sciences, Duesseldorf, Germany

[2] Professorship for Exercise Biology, School of Medicine and Health, Technical University of Munich, Germany

[3] Department of Sport and Management, IST University of Applied Sciences, Duesseldorf, Germany

† Shared last authorship

*Corresponding author: thavers@ist-hochschule.de

Journal name: Sports Medicine

Supplementary Material

## Contents

**Online Supplementary Appendix 1:** Methods – Systematic Literature Search

**Online Supplementary Appendix 2:** Prisma Flow Chart

**Online Supplementary Appendix 3:** Inclusion and exclusion criteria based on the PICO framework

**Online Supplementary Appendix 4:** Results Table Human Interventions (separate file)

**Online Supplementary Appendix 5:** Results Table Animal Interventions (separate file)

**Online Supplementary Appendix 6:** Effects of Global Muscle Hypertrophy on Fat Mass Based on Body Mass Index (A), Age (B), and Resistance Training Experience (C)

**Online Supplementary Appendix 7:** Effects of Global Muscle Hypertrophy on Glucose Homeostasis Based on Body Mass Index (A) and Age (B)

**Online Supplementary Appendix 1:** Methods – Systematic Literature Search

**Search strategy**

To identify publications that investigated muscle hypertrophy and its anti-diabetes and anti-obesity effects, we carried out a systematic literature search which was conducted according to the PICO framework [1]. Specifically, our research question was: In animal models or human beings (population), how does any form of induced muscle hypertrophy (intervention) affect glucose homeostasis and/or fat mass (outcome)? To answer this question, the scientific databases PubMed/MEDLINE, SPORTDiscus, and Scopus were systematically searched on September 17, 2024, to identify all relevant studies according to our PICO framework. We identified all English-language peer-reviewed research studies that induced any form of global muscle hypertrophy (i.e., exercise, pharmacology, or a combination) in humans as well as in transgenic animal models and reported simultaneously changes in glucose metabolism (i.e., HbA1c or blood glucose) or fat mass. The following systematic research strategy was applied: “(human OR pig OR mouse OR rat) AND ("hypertrophy" OR "muscle growth" OR "muscle fiber growth" OR "lean body mass") AND (("body composition" OR "fat mass" OR "body fat" OR "adipose tissue") OR ("HbA1c" OR "fasting blood glucose" OR "glycemic control" OR "glucose control" OR "glycated hemoglobin"))”. The reference list of the retrieved articles was also screened, as well as the reference lists of previously published reviews. See **Online Supplementary Appendix 2** for the Prisma flow chart [1]. The screening process was performed using online software (https://www.rayyan.ai/).

**Eligibility criteria**

Eligibility criteria are provided in detail in **Online Supplementary Appendix 3.** Studies involving animals (i.e., pig, mouse, rat) or humans ≥18 years of age who were either healthy, sarcopenic, diabetic, or obese were included if any form of positive global muscle hypertrophy was induced and a glucose metabolism parameter or a fat mass parameter (i.e., kg or body fat percentage) were reported (i.e., at least one of the two). HbA1c and blood glucose parameters were used as the glucose parameters of choice. If only body fat percentage and body weight were available, fat mass in kilograms was calculated (body weight x body fat percentage). If no positive hypertrophic response was available, the study was excluded. Studies that included children or adolescents, individuals with comorbidities other than those described, animal models other than those described, and non-English language studies were excluded. For muscle mass outcomes concerning the human population, we included lean body mass, fat-free mass, or skeletal muscle mass measured by dual-energy x-ray (DEXA) or bioelectrical impedance analysis (BIA), while muscle mass outcomes with respect to the animal studies included muscle weight by dissection and DEXA lean mass.

**Study selection and data extraction**

Based on the eligibility criteria, title/abstract and full-text screening were performed. Data from human interventions and animal studies were extracted separately. From human interventions, we extracted information on author (year), participant information (i.e., number of participants, age, sex, resistance training experience), treatment (i.e., resistance training, pharmacological, combination of both), global muscle mass measure, glucose parameter, and fat mass. We calculated the percentage change in LBM, FFM, or SKM from baseline to post-measurement (pre-post comparison) also for FM and glucose parameters.

From animal studies, we extracted information on author (year), animal-related information (i.e., animal species, lineage), treatment (i.e., resistance training, pharmacological intervention, transgenic modification, or a combination), muscle mass measure (i.e., muscle weight, DEXA), fat mass measure (i.e., fat weight, DEXA), and blood glucose from the intervention and control conditions. Because longitudinal hypertrophic interventions are not sufficiently feasible in animal models, we compared the intervention condition (i.e., where the specific treatment was applied) to the control condition. Specifically, we compared the muscle/fat variable of interest of the experimental condition to the muscle/ fat variable of the control condition. Furthermore, we also compared the muscle/fat variable of interest relative to the body weight of the animal. In cases where the parameter of interest was only available in graphs/figures, we extracted the data via online software (https://automeris.io/WebPlotDigitizer/). The percentage differences between the intervention and control conditions were calculated for muscle mass, fat mass, and glucose parameters.

**Data analysis**

Data analysis was performed separately for human and animal interventions. The overall mean percentage change in muscle mass (MM), fat mass (FM), HbA1c, and blood glucose concentration was calculated. We then categorized the human and animal studies into domains of interest: (1) fat mass domain and (2) glucose domain. For each domain, we calculated the overall percentage change in fat mass or glucose (HbA1c and blood glucose combined), along with the corresponding percentage change in muscle mass for each species. Moreover, we calculated the mean percentage changes (and standard deviations) for each treatment condition: for humans (pharmaceutical, resistance training, combined) and for animals (pharmaceutical, resistance training, combined, transgenic). Where applicable, linear regressions (treatment: pharmaceutical, treatment: overall minus pharmaceutical) were calculated for each domain of interest, and the results were displayed in scatterplots. Next, we created subgroups based on age (18–39 years, 40–64 years, ≥ 65 years), body mass index (BMI) (normal weight: 18.5–24.9 kg/m², overweight: 25.0–29.9 kg/m², obesity: ≥ 30.0 kg/m²), and resistance training experience (inexperienced, experienced) for human intervention studies. Linear regressions were also performed for each subgroup and illustrated in scatterplots.

**Online Supplementary Appendix 2:** Prisma flow chart [1]

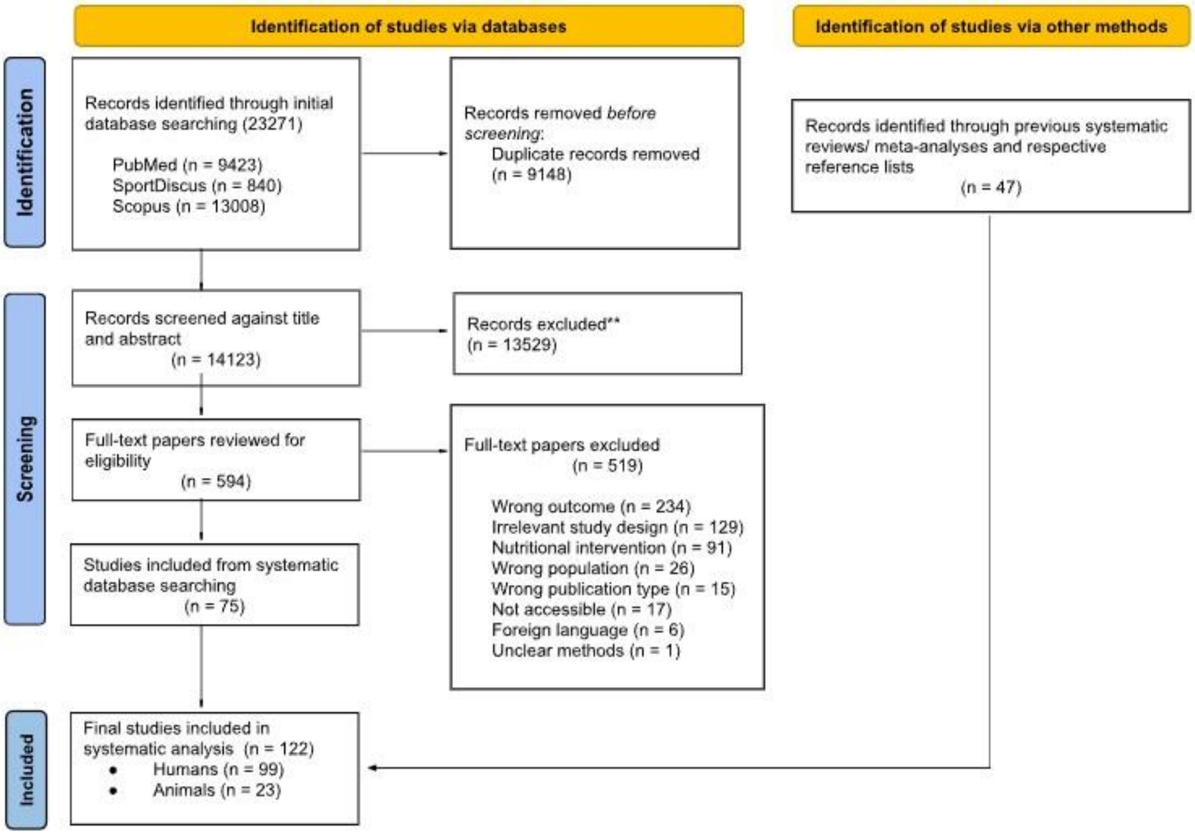

**Online Supplementary Appendix 3:** Inclusion and exclusion criteria based on the PICO framework

| **Inclusion criteria** | **Exclusion criteria** |
|---|---|
| Population<br>• Animal models: pigs, mice, rats<br>• Human beings<br>  o ≥18 years old<br>  o Healthy or no other disease condition except than sarcopenia, diabetes, obesity) | Population<br>• Animals other than pigs, mice, and rats<br>• < 18 years of age<br>• Individuals with or at risk of co-morbidities such as: cardiovascular disease, osteoarthritis, osteopenia)<br>• Hypogonadism or patients who are hypogonadal and receiving growth hormone therapy or hormone replacement therapy<br>• Individuals with injuries (e.g., fractures)<br>• Hospitalized individuals |
| Intervention<br>Human beings:<br>• Any intervention that induces global muscle hypertrophy from pre-to post-test<br>  o Resistance training<br>  o Pharmaceutical treatment (e.g., growth hormone or testosterone administration, etc.)<br>  o Combination of resistance training and pharmaceutical treatment<br>• Global muscle hypertrophy parameters: fat-free mass, fat-free and bone-free mass or lean (body) mass<br>• Eligible measurement instruments:<br>  o Bioelectrical impedance analysis (BIA)<br>  o Dual-Energy-X-ray Absorptiometry (DEXA)<br>Animal models:<br>• Any intervention that induces global muscle hypertrophy<br>  o Resistance training<br>  o Pharmaceutical treatment (e.g., hormones, beta antagonists etc.)<br>  o Transgenic modification (e.g., knock-out)<br>• Global muscle hypertrophy parameters:<br>  o Fat-free mass, fat-free and bone-free mass or lean (body) mass<br>  o Total muscle weight<br>• Eligible measurement instruments:<br>  o Dual-Energy-X-ray Absorptiometry (DEXA)<br>  o Dissection | Intervention<br>• Any form of exercise intervention other than high load (>60%1RM) or low load (<60%1RM) resistance training<br>• Any form of exercise intervention that interferes with an increase in global muscle hypertrophy (e.g., endurance training, yoga, Pilates, etc.)<br>• Caloric deficit studies and other dietary modifications<br>• Dietary supplementation<br>• Drugs that suppress muscle hypertrophy<br>• Other measures of hypertrophy: e.g. hydrostatic weighing<br>• No muscle hypertrophy<br><br>Animal models:<br>• Physical activity other than resistance training (e.g. running, treadmill, etc.)<br>• Supplementation |
| Comparison<br>- | Comparison<br>- |
| Outcome<br>Human beings:<br>• Glucose metabolism parameter<br>  o Glycosylated hemoglobin (HbA1c) | Outcome<br>Human beings:<br>• No measure of HbA1c, fasting blood glucose or fat mass parameters |

| | |
|---|---|
| ○ Fasting blood glucose<br>• Fat mass parameter<br>○ Fat mass in kg or body fat percentage<br>○ Fat mass measured by BIA or DEXA<br>• Calculation of the percentage change from pre-to post test<br>Animal models:<br>• Glucose metabolism parameter<br>○ Glycosylated hemoglobin (HbA1c)<br>○ Blood glucose<br>• Fat mass parameter<br>○ Fat mass in kg<br>○ Fat mass measured by muscle weighing or DEXA | Animal models:<br>• No measurements of HbA1c or any blood glucose or fat pad/mass weight parameter<br>• Percentage difference between experimental and control animals not possible |
| Study design<br>Human beings:<br>• Reported in English<br>• Longitudinal studies<br>• Cross-over studies<br>• Study groups needed to be extractable<br><br>Animal models:<br>• Reported in English<br>• Data from experimental group(s) and control group(s) (e.g. wild-type animal) should be extractable | Study design<br>Human beings:<br>• Observational studies (e.g., retrospective, prospective)<br>• Acute interventions<br>• Reviews of any kind<br><br>Animal models:<br>• Longitudinal studies (e.g., pre-post comparisons)<br>• Reviews of any kind<br>• Missing control group |

**Online Supplementary Appendix 6:** Effects of Global Muscle Hypertrophy on Fat Mass Based on Body Mass Index (A), Age (B), and Resistance Training Experience (C)

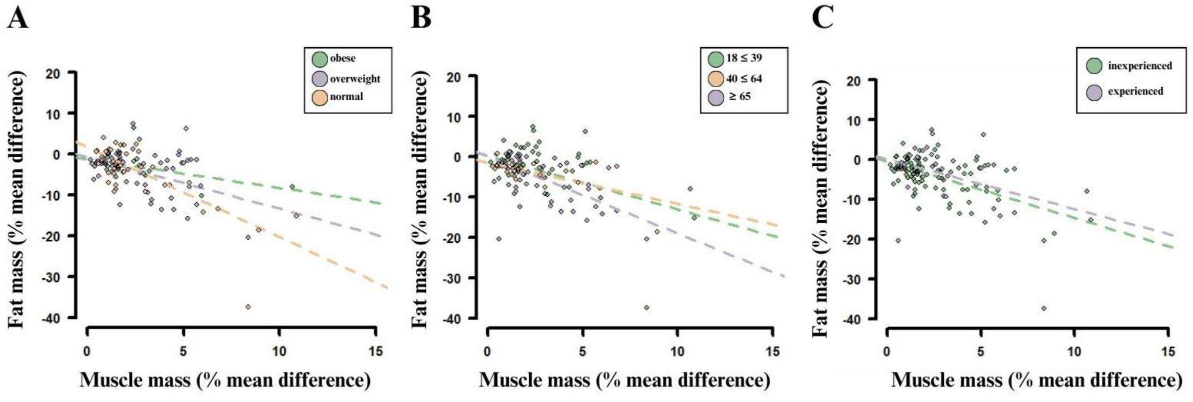


**Description:** **A** represents the effect of global muscle hypertrophy on fat mass based on body mass index (kg/$m^2$), here classified into normal weight (k = 33), overweight (k = 71), and obese (k = 19). **B** represents the relationship between muscle mass and fat mass based on age (y), here classified into 18 ≤ 39 years (k = 53), 40 ≤ 64 years (k = 25), and ≥ 65 years (k = 50). **C** represents the relationship between muscle mass and fat mass based on resistance training experience (experienced: k = 21; inexperienced: k =109).

| **Linear regression analyses** | **Body Mass Index (kg/m²)** | **Age (y)** | **Resistance training experience (Inexperienced/experienced)** |
|---|---|---|---|
| **Subgroup 1** | Normal weight | 18 ≤ 39 years | Inexperienced |
| Function | y = -2.202 * x + 1.588 | y = -1.326 * x + 0.295 | y = -1.428 * x -0.44 |
| Correlation (r) | -0.70 | -0.43 | -0.53 |
| Regression ($r^2$) | 0.49 | 0.18 | 0.38 |
| P -value | **< 0.001** | **< 0.05** | **< 0.001** |
| **Subgroup 2** | Overweight | 40 ≤ 64 years | Experienced |
| Function | y = -1.271 * x -0.667 | y = -1.035 * x -1.39 | y = -1.246 * x + 0.062 |
| Correlation (r) | -0.47 | -0.63 | -0.45 |
| Regression ($r^2$) | 0.21 | 0.40 | 0.20 |
| P -value | **< 0.001** | **< 0.001** | **<0.05** |
| **Subgroup 3** | Obese | ≥ 65 years | |
| Function | y = -0.705 * x -1.254 | y = -1.902 * x + 0.083 | |
| Correlation (r) | -0.51 | -0.70 | |
| Regression ($r^2$) | 0.26 | 0.50 | |
| P -value | **<0.05** | **< 0.001** | |

**Online Supplementary Appendix 7:** Effects of Global Muscle Hypertrophy on Glucose Homeostasis Based on Body Mass Index (A) and Age (B)

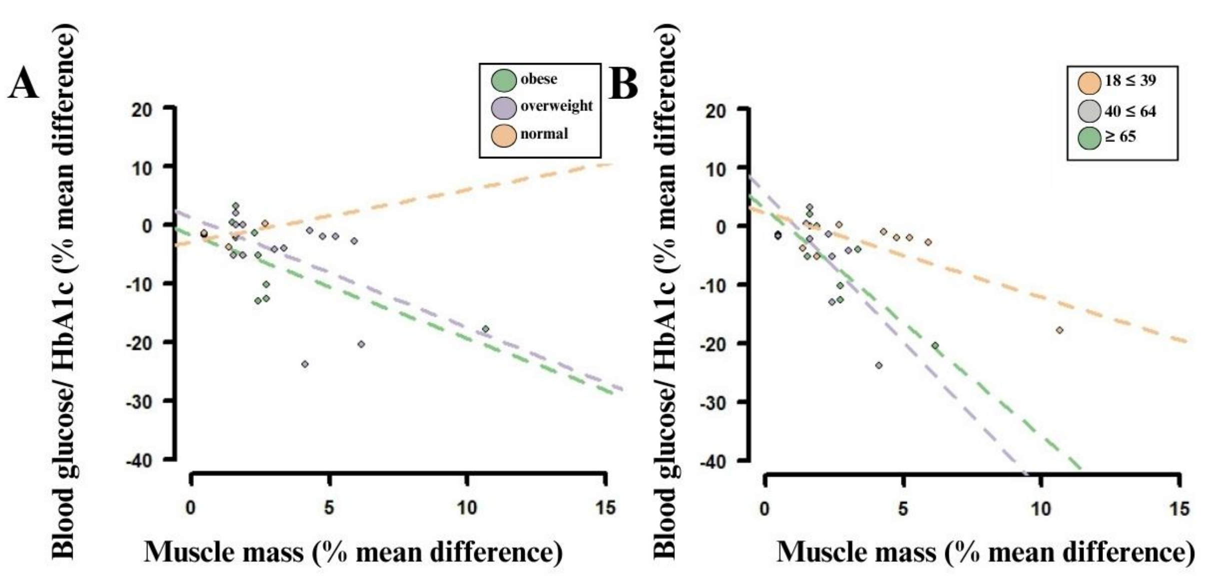


**Description:** **A** represents the effect of global muscle hypertrophy on glucose homeostasis based on body mass index (kg/m$^2$), here classified into normal weight (k = 3), overweight (k = 14), and obese (k = 14). **B** represents the relationship between muscle mass and fat mass based on age (y), here classified into 18 ≤ 39 years (k = 8), 40 ≤ 64 years (k = 11), and ≥ 65 years (k = 11).

| **Linear regression analyses** | **Body Mass Index (kg/m²)** | **Age (y)** |
|---|---|---|
| **Subgroup 1** | Normal weight | 18 ≤ 39 years |
| Function | f(x) = -2.946737 + 0.8895318 * x | f(x) = 2.279765 + -1.438524 * x |
| Correlation (r) | 0.49 | -0.74 |
| Regression (r²) | 0.24 | 0.55 |
| P -value | 0.672 | **< 0.05** |
| **Subgroup 2** | Overweight | 40 ≤ 64 years |
| Function | f(x) = 1.223665 + -1.87699 * x | f(x) = 5.492282 + -5.062132 * x |
| Correlation (r) | -0.426 | -0.72 |
| Regression (r²) | 0.181 | 0.51 |
| P -value | 0.129 | **< 0.05** |
| **Subgroup 3** | Obese | ≥ 65 years |
| Function | f(x) = -1.567432 + -1.775415 * x | f(x) = 2.798254 + -3.861566 * x |
| Correlation (r) | -0.679 | -0.81 |
| Regression (r²) | 0.461 | 0.65 |
| P -value | **<0.05** | **< 0.05** |

# References

1. Page MJ, McKenzie JE, Bossuyt PM, Boutron I, Hoffmann TC, Mulrow CD, et al. The PRISMA 2020 statement: an updated guideline for reporting systematic reviews. BMJ. 2021;372:n71. doi:10.1136/bmj.n71.
